# Milk Diet

**Published:** 1884-11

**Authors:** 


### MILK DIET.

THE greater the advance in knowledge of the science of medicine, and of the care and treatment of the human body to preserve its health, the more importance is attached to the use of milk for food, and to its healing properties when administered in illness. Milk contains nourishment for every part of the body. Bone, muscle, brain, and flesh can be sustained in fine condition by milk diet. The human frame is healthier when fed by variety in food; yet a certain quantity of milk should be taken every day by every person. There never was an article of diet so subject to whims and ignorant prejudices as milk. Some dislike it, others it makes "sleepy," "bilious," or "headachy." It affects the bowels of many, and is constipating to others. The persons who dislike milk, or with whom it "does not agree," are invariably the ones who require it, and whom it would probably rejuvenate did they so prepare it as to make it palatable and suitable to their peculiar constitutions. Milk diluted one-third with lime-water will not cause any one biliousness or headache, and if taken regularly will so strengthen the stomach as to banish these disorders. It may be taken with acid of some kind when it does not easily digest. The idea that milk must not be eaten with pickles is not an intelligent one, as milk curdles in the stomach almost as soon as it is swallowed. When milk is constipating, as it is frequently found to be by persons who drink freely of it in the country in summer time, a little salt sprinkled in each glassful will prevent the difficulty. As milk is so essential to the health of our bodies, it is as well to consider when to take it, as how. It is a mistake to drink milk between meals. After finishing each meal a goblet of pure milk may be drank, and if any one wishes to grow fleshy a bowl of bread and milk may be taken before retiring at night. In cases of fever and summer complaint milk is now given with excellent results. The idea that milk is "feverish" has exploded, and it is now the physician's great reliance in bringing through typhoid patients, or those in too low a state to be nourished by solid food. It is a mistake to scrimp the milk-pitcher. Take more milk, and buy less meat. Look to your milkman, have large-sized, well-filled milk-pitchers on the table each meal, and you will also have sound flesh and light doctor's bills.

But one thing is of the first importance, viz., that the milk must be pure and sweet. Milk which comes from badly-fed cows, or which has been kept till it becomes stale, is worse than worthless as food. There are not many stomachs which comfortably digest milk which is twenty-four hours old. Pure, new milk, warm from the cow, if taken in moderation, will not constipate nor occasion heaviness or headache. To some persons it may induce a trifling sense of fullness at first, but that will pass off in three or four days. Thousands of cases of dyspepsia, chronic, or habitual headache and gastric disturbance may be cured by the use of pure, sweet milk as the sole beverage and chief food.

But the most valuable preparation of milk, for imperfect digestion and the multitude of ills that result from dyspepsia, undoubtedly is "kumiss." The great drawback to its more general use is that a taste for it has usually to be acquired. It is not often relished at first, but one soon becomes fond of it. As a rule, "kumiss" agrees with the weakest stomach, and is the most easily digested of all foods. It is both food and medicine.

